# Role of Transient Receptor Potential Vanilloid 1 in Electroacupuncture Analgesia on Chronic Inflammatory Pain in Mice

**DOI:** 10.1155/2017/5068347

**Published:** 2017-12-12

**Authors:** Jun Yang, Ching-Liang Hsieh, Yi-Wen Lin

**Affiliations:** ^1^Department of Acupuncture, China Medical University Hospital, Taichung 40402, Taiwan; ^2^College of Chinese Medicine, Graduate Institute of Acupuncture Science, China Medical University, Taichung 40402, Taiwan; ^3^College of Chinese Medicine, Graduate Institute of Integrated Medicine, China Medical University, Taichung 40402, Taiwan; ^4^Department of Chinese Medicine, China Medical University Hospital, Taichung 40402, Taiwan; ^5^Research Center for Chinese Medicine & Acupuncture, China Medical University, Taichung 40402, Taiwan

## Abstract

Chronic inflammatory pain may result from peripheral tissue injury or inflammation, increasing the release of protons, histamines, adenosine triphosphate, and several proinflammatory cytokines and chemokines. Transient receptor potential vanilloid 1 (TRPV1) is known to be involved in acute to subacute neuropathic and inflammatory pain; however, its exact mechanisms in chronic inflammatory pain are not elucidated. Our results showed that EA significantly reduced chronic mechanical and thermal hyperalgesia in the chronic inflammatory pain model. Chronic mechanical and thermal hyperalgesia were also abolished in TRPV1^−/−^ mice. TRPV1 increased in the dorsal root ganglion (DRG) and spinal cord (SC) at 3 weeks after CFA injection. The expression levels of downstream molecules such as pPKA, pPI3K, and pPKC increased, as did those of pERK, pp38, and pJNK. Transcription factors (pCREB and pNF*κ*B) and nociceptive ion channels (Nav1.7 and Nav1.8) were involved in this process. Inflammatory mediators such as GFAP, S100B, and RAGE were also involved. The expression levels of these molecules were reduced in EA and TRPV1^−/−^ mice but not in the sham EA group. Our data provided evidence to support the clinical use of EA for treating chronic inflammatory pain.

## 1. Introduction

Chronic pain is a pathological condition that persists for more than 3–6 months since the onset of pain in human beings [1]. The chronic pain model produces a secondary mechanical hyperalgesia in the central nervous system (CNS). Epidemiological studies indicate that approximately 9%–64% of people worldwide are afflicted by chronic pain [2]. Chronic inflammatory pain arises when inflammatory mediators are not neutralized, and the overexpression of proinflammatory cytokines and chemokines further leads to peripheral and central sensitization [3]. Chronic inflammatory pain may result from peripheral injury sites, spinal cord, or central brain and is usually difficult to cure. Patients with chronic pain may be occasionally relieved by opioid administration, even though other treatments are ineffective [4]. Chronic pain without curative treatment has increased the mortality rate in patients with heart disease, depression, anxiety, and sleep disturbances [5, 6]. Patients afflicted by chronic pain always perform reduced exercise and physical activities, and exercise is an intervention to alleviate pain [7]. Chronic inflammatory pain is defined as nociceptive and neuropathic pain caused by inflamed tissue with activated nociceptors and malfunctioning nerves, respectively [8].

Transient receptor potential (TRP) family has six transmembrane domains that can be further categorized into seven subtypes: TRPV (vanilloid), TRPA (ankyrin), TRPC (canonical), TRPM (melastatin), TRPML (mucolipin), TRPP (polycystin), and TRPN (no mechanoreceptor potential C) [9]. TRPV1 channels can respond to heat, touch, pain, and physical and chemical stimuli [9]. They can recognize sensory inputs from peripheral nerve terminals including painful signals. The activation of TRPV1 channels subsequently induces cation influx, especially that of Ca^2+^, and further activates signaling pathways. TRPV1 is well known as a nociceptor in peripheral sensory neurons [10]. TRPV1, also known as a capsaicin receptor, is a nonselective cation channel that can be activated by mechanical, thermal (temperature more than 43°C), and chemical stimuli. The activation of TRPV1 leads to mechanical, thermal, and burning sensations. In some pathological conditions, this may pain. TRPV1 is primarily found in peripheral nociceptive neurons for pain transduction and is also reportedly involved in integrating painful stimuli [11, 12].

Acupuncture was first reported to relieve pain in the 1970s by Johnson [13]. Evidence-based studies have suggested that electroacupuncture (EA) can be used to treat learning and memory impairment in rats with cerebral ischemia-reperfusion injury [14], epilepsy [15], body weight control [16], and pain [17–20]. In addition, several studies have suggested that acupuncture can significantly increase the release of endogenous opiates [21], serotonin [22], and adenosine [23] to alleviate nociception. The major mechanism underlying acupuncture analgesia is the release of opiates in CNS. The aim of the present study is to examine the role of TRPV1 in chronic inflammatory pain and to test the expression of TRPV1 and related downstream molecules after inducing chronic inflammatory pain in the peripheral dorsal root ganglion (DRG) and central spinal cord (SC).

## 2. Methods

### 2.1. Animals

Serial experiments were conducted on C57/B6 male mice (ages 8 to 12 weeks) purchased from BioLASCO Co., Ltd, Taipei, Taiwan. Mice were randomly subdivided into 5 groups (*n* = 8 per group): (1) Control, (2) CIP (CFA-induced chronic inflammatory pain), (3) 2 Hz EA (CFA-induced chronic inflammatory pain with 2 Hz EA), (4) sham EA (CFA-induced chronic inflammatory pain with sham EA), and (5) TRPV1^−/−^ (CFA-induced chronic inflammatory pain in TRPV1^−/−^ mice). The sample size required for an alpha of 0.05 and a power of 80% is 8 animals per group. After arrival, mice were housed in a 12/12 h light/dark cycle with ad libitum water and food. All procedures were approved by the Institute of Animal Care and Use Committee of China Medical University (permit number 2016-061) and conducted in accordance with the Guide for the use of Laboratory Animals by the National Research Council and the ethical guidelines of the International Association for the Study of Pain. The mice suffering was minimized with 1% isoflurane. The laboratory workers were blinded to treatment allocation during the experiments and analysis.

### 2.2. Chronic Inflammatory Pain Induction

Mice were anesthetized with 1% isoflurane and given a single injection of 20 *μ*l saline (pH 7.4, buffered with 20 mM HEPES) or CFA (75% in saline, 0.5 mg/ml heat-killed* M. tuberculosis* [Sigma, St. Louis, MO]) in the plantar surface of the hind paw to induce chronic intraplantar inflammation [19]. Behaviour tests are conducted every 3 days after induction of chronic inflammatory pain.

### 2.3. Electroacupuncture Treatment

Electroacupuncture (EA) was applied using stainless steel needles (0.5′′ inch, 32 G, Yu-Kuang, Taiwan) inserted into the muscle layer to a depth of 2-3 mm at the bilateral ST36 acupuncture point. The ST36 acupuncture point is located on the tibialis anterior muscle, approximately 1/6 of the distance from the patella to the lateral malleolus. Disposable needles with a diameter of 0.30 mm and a length of 13 mm (Yu-Kuang Acupuncture Instrument Co., Taiwan) were inserted into the muscle layer at both ST36 acupuncture points to a depth of 2-3 mm. EA was administered at days 15, 18, and 21 after the CFA injection. A stimulator (Trio 300, Ito, Japan) delivered 100-*μ*s square pulses of 1 mA for 15 min at 2 Hz. EA was applied at nonacupoint to be set as sham control group (upper gluteal muscle).

### 2.4. Animal Behaviour of Mechanical and Thermal Hyperalgesia

Mechanical and thermal sensitivities were tested at 3 days after intraplantar injection of CFA. All experiments were performed at room temperature (approximately 25°C) and the stimuli were applied only when the animals were calm but not sleeping or grooming. Mechanical sensitivity was measured by testing the force of responses to stimulation with 3 applications of electronic von Frey filaments (North Coast Medical, Gilroy, CA, USA). At 1 h after mechanical testing, thermal pain was measured with 3 applications using Hargraves' test IITC analgesiometer (IITC Life Sciences, Woodland Hills, CA, USA) [19].

### 2.5. Immunoblotting Assay

After animal behaviour test (at day 21), mice DRG and SC dorsal horn were immediately excised to extract proteins. Total proteins were prepared by homogenized tissue in lysis buffer containing 50 mM Tris-HCl pH 7.4, 250 mM NaCl, 1% NP-40, 5 mM EDTA, 50 mM NaF, 1 mM Na3VO4, 0.02% NaN3, and 1x protease inhibitor cocktail (AMRESCO). The extracted proteins (30 *μ*g per sample assessed by BCA protein assay) were subjected to 8% SDS-Tris glycine gel electrophoresis and transferred to a PVDF membrane. The membrane was blocked with 5% nonfat milk in TBS-T buffer (10 mM Tris pH 7.5, 100 mM NaCl, 0.1% Tween 20), incubated with primary antibody against TRPV1 (~95 kDa, 1 : 1000, Alomone, Israel), pPKA (~51 kDa, 1 : 1000, Millipore, USA), pPI3K (~125 kDa, 1 : 1000, Millipore, USA), pPKC (~80–82 kDa, 1 : 1000, Millipore, USA), pERK1/2 (~42–44 kDa, 1 : 1000, Millipore, USA), pp38 (~41 kDa, 1 : 1000, Millipore, USA), pJNK (~42 kDa, 1 : 1000, Millipore, USA), pAkt (~60 kDa, 1 : 1000, Millipore, USA), pmTOR (~60 kDa, 1 : 500, Millipore, USA), pCREB (~43 kDa, 1 : 1000, Millipore, USA), pNF*κ*B (~65 kDa, 1 : 1000, Millipore, USA), Nav1.7 (~230 kDa, 1 : 1000, Millipore, USA), Nav1.8 (~220 kDa, 1 : 1000, Millipore, USA), GFAP (~50 kDa, 1 : 1000, Millipore, USA), S100B (~10–15 kDa, 1 : 1000, Millipore, USA), and RAGE antibody (~48 kDa, 1 : 1000, Millipore, USA), in TBS-T with 1% bovine serum albumin, and incubated for 1 hour at room temperature. Peroxidase-conjugated anti-rabbit antibody (1 : 5000) was used as a secondary antibody. The bands were visualized by an enhanced chemiluminescence substrate kit (PIERCE) with LAS-3000 Fujifilm (Fuji Photo Film Co. Ltd). Where applicable, the image intensities of specific bands were quantified with NIH ImageJ software (Bethesda, MD, USA) [19].

### 2.6. Statistical Analysis

All statistic data are presented as the mean ± standard error. Statistical significance between control, CIP, 2 Hz EA, sham EA, and TRPV1^−/−^ groups was tested using the ANOVA test, followed by a post hoc Tukey's test (*p* < 0.05 was considered statistically significant).

## 3. Results

**Figure 1:**
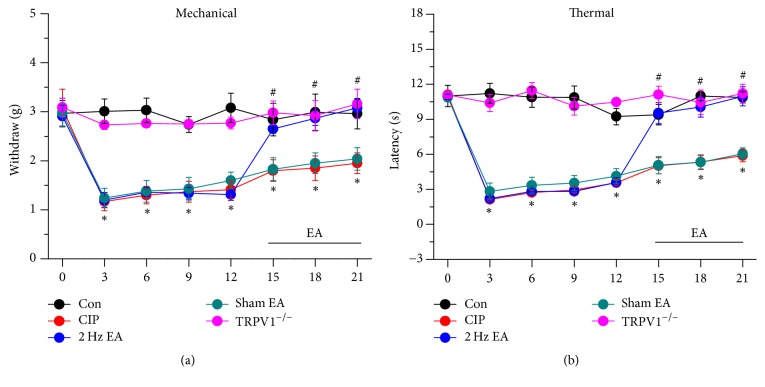
Mechanical and thermal withdrawal thresholds in each group of mice. Normal saline injection (Con group, *n* = 8), CIP (CFA-induced chronic inflammatory pain), 2 Hz EA (CFA-induced chronic inflammatory pain with 2 Hz EA), sham EA (CFA-induced chronic inflammatory pain with sham EA), and TRPV1^−/−^ (CFA-induced chronic inflammatory pain in TRPV1^−/−^ mice). ^*∗*^*p* < 0.05 versus Con. ^#^*p* < 0.05 versus CIP group.

Acupuncture has been used for thousands of years to manage pain, albeit with unclear mechanisms. We used mechanical and thermal pain behaviour to examine the role of TRPV1 in EA analgesia on CFA-induced chronic inflammatory pain model. Mechanical hyperalgesia was not induced at day 21 in control mice injected with normal saline (Figure 1(a), mechanical force = 2.96 ± 0.31 g, *n* = 8). Chronic mechanical hyperalgesia was induced after CFA injection on day 21 (Figure 1(a), 1.95 ± 0.21 g, *n* = 8). We then identified that 2 Hz EA significantly attenuated CFA-induced chronic mechanical hyperalgesia on day 21 (Figure 1(a), 3.08 ± 0.17 g, *n* = 8). With acupoint specificity, sham EA did not alter mechanical hyperalgesia (Figure 1(a), 2.04 ± 0.23 g, *n* = 8). Our results further suggested that TRPV1 was crucial for chronic pain because chronic mechanical hyperalgesia was not induced in TRPV1^−/−^ mice (Figure 1(a), 3.16 ± 0.3 g, *n* = 8). We further investigated if thermal hyperalgesia was similarly affected. Injection of normal saline did not induce thermal hyperalgesia (Figure 1(b), thermal latency = 10.87 ± 0.48 g, *n* = 8). Injection of CFA reliably induced chronic thermal hyperalgesia (Figure 1(b), 5.89 ± 0.52 g, *n* = 8) and it was further reduced by 2 Hz EA stimulation (Figure 1(b), 10.96 ± 0.81 g, *n* = 8). The effect was not observed in the sham-operated group (Figure 1(b), 6.06 ± 0.48 g, *n* = 8). Moreover, thermal hyperalgesia induced by CFA was not seen in TRPV1^−/−^ mice (Figure 1(b), 11.22 ± 0.79 g, *n* = 8). The aforementioned results indicate that EA dramatically decreased both chronic mechanical and thermal hyperalgesia in a mouse chronic inflammatory pain model.

**Figure 2:**
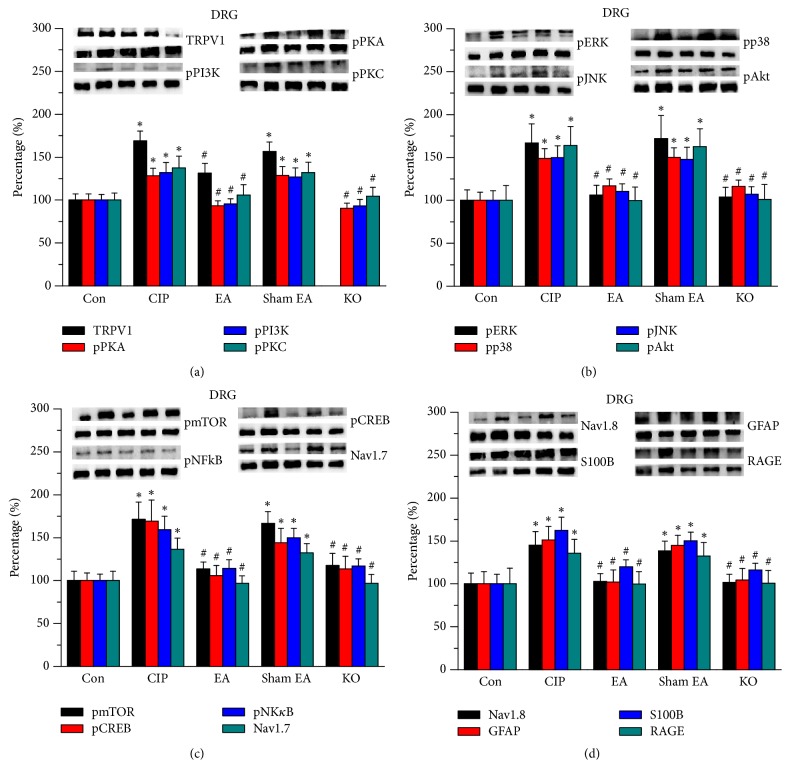
Expression levels of TRPV1-associated signaling pathway in mice DRG. (a) TRPV1, pPKA, pPI3K, and pPKC. (b) pERK, pp38, pJNK, and pAkt. (c) pmTOR, pCREB, pNF*κ*B, and Nav1.7. (d) Nav1.8, GFAP, S100B, and RAGE expression levels in tissues from Con, CIP, 2 Hz EA, sham EA, and TRPV1^−/−^ groups (from left to right). Con: control; CIP: chronic inflammatory pain; EA: electroacupuncture; sham EA: sham electroacupuncture; KO: TRPV1 knockout mice. ^*∗*^*p* < 0.05 versus Con. ^#^*p* < 0.05 versus CUP group. The western blot bands at the top show the target protein. The lower bands are internal controls (*β*-actin or *α*-tubulin).

Subsequently, we used western blot analysis to determine if chronic inflammatory pain could change the protein level of TRPV1 and the associated downstream molecules. Our data suggested that TRPV1 was expressed in DRG in control mice (Figure 2(a), 100.1 ± 7.31%, *n* = 8) and then increased in chronic inflammatory pain in mice (Figure 2(a), 168.89 ± 11.6%, *n* = 8). In addition, 2 Hz EA significantly reduced the overexpression of TRPV1 (Figure 2(a), 131.23 ± 11.48%, *n* = 8). Sham EA treatment did not alter this observation (Figure 2(a), 156.69 ± 10.88%, *n* = 8).

We then addressed the effects of EA on TRPV1 and the associated downstream protein kinases. We showed that pPKA, pPI3K, and pPKC levels increased in chronic inflammatory pain in mice DRG (Figure 2(a), 128.3 ± 8.86%, 132.13 ± 11.59%, and 137.38 ± 13.93%, *n* = 8) and then reversed by EA (Figure 2(a), 93.51 ± 5.59%, 95.28 ± 6.33%, and 105.94 ± 11.89%, *n* = 8) but not by the sham treatment (Figure 2(a), 128.55 ± 10.41%, 126.95 ± 10.45%, and 132.02 ± 12.15%, *n* = 8). The overexpression of such protein kinases decreased in TRPV1^−/−^ mice (Figure 2(a), 90.21 ± 6.36%, 92.98 ± 7.89%, and 104.57 ± 10.45%, *n* = 8). We then examined the downstream protein kinases in all groups. We showed that pERK, pp38, pJNK, and pAkt signaling pathways were upregulated in chronic inflammatory pain in mice (Figure 2(b), 167.08 ± 22.08%, 148.74 ± 11.5%, 149.67 ± 13.82%, and 163.93 ± 22.15%, *n* = 8). The overexpression of these molecules was further reduced by EA (Figure 2(b), 106.15 ± 11.31%, 117.05 ± 7.77%, 110.69 ± 8.66%, and 99.77 ± 15.66%, *n* = 8) but not by the sham treatment (Figure 2(b), 171.91 ± 26.85%, 150.14 ± 11.28%, 147.82 ± 14.19%, and 162.47 ± 21.12%, *n* = 8). All signals were decreased to control level in TRPV1^−/−^ mice (Figure 2(b), 103.74 ± 11.65%, 116.09 ± 7.58%, 107.08 ± 8.87%, and 101.15 ± 17.55%, *n* = 8). Similar results were also observed in pmTOR expression (Figure 2(c), *n* = 8).

We further illustrated that the transcription factors pCREB and pNK*κ*B also increased in chronic inflammatory pain in mice (Figure 2(c), 169.32 ± 24.39%, and 159.16 ± 16.03%, *n* = 8), which was then attenuated by EA (Figure 2(c), 105.93 ± 11.64% and 113.50 ± 8.19%, *n* = 8) but not by the sham treatment (Figure 2(c), 136.36 ± 12.99% and 143.96 ± 17.4%, *n* = 8). The overexpression of pCREB and pNK*κ*B was not observed in TRPV1^−/−^ mice (Figure 2(c), 13.67 ± 14.7%, and 117.48 ± 14.24%, *n* = 8). Furthermore, the expression levels of the nociceptive channels Nav1.7 and Nav1.8 increased in chronic pain in mice (Figures 2(c) and 2(d), 135.86 ± 12.99% and 139.16 ± 16.03%, *n* = 8), which were reduced by EA and TRPV1 deletions but not in the sham treated group (Figures 2(c) and 2(d), *n* = 8).  Figure 2(d) shows that the levels of GFAP (satellite cell marker in DRG), S100B, and RAGE were upregulated in chronic inflammatory pain in mice (145.18 ± 15.85, 151.34 ± 15.78%, and 136.26 ± 13.29%). EA (102.96 ± 8.83%, 119.87 ± 8.22%, and 102.08 ± 14.19%) and TRPV1 deletion (104.39 ± 13.37%, 116.14 ± 7.95%, and 101.69 ± 9.42%) significantly reduced inflammatory biomarkers but not in the sham treated group (144.5 ± 11.7%, 150.21 ± 9.9%, and 132.2 ± 16.06%).

**Figure 3:**
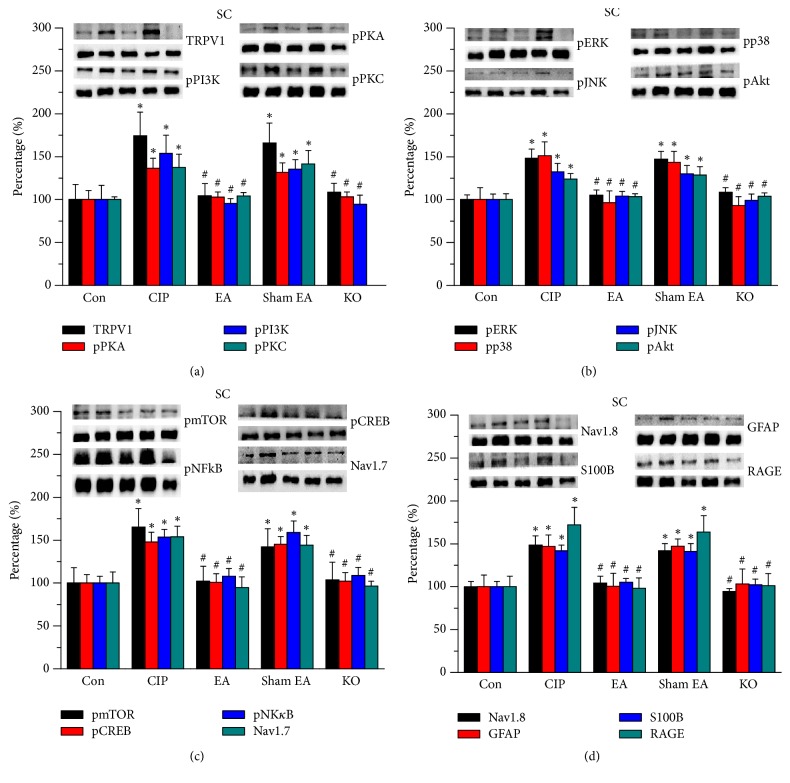
Expression levels of TRPV1-associated signaling pathway in mice SC. (a) TRPV1, pPKA, pPI3 K, and pPKC. (b) pERK, pp38, pJNK, and pAkt. (c) pmTOR, pCREB, pNF*κ*B, and Nav1.7. (d) Nav1.8, GFAP, S100B, and RAGE expression levels in tissues from Con, CIP, 2 Hz EA, sham EA, and TRPV1^−/−^ groups (from left to right). Con: control; CIP: chronic inflammatory pain; EA: electroacupuncture; sham EA: sham electroacupuncture; KO: TRPV1 knockout mice. ^*∗*^*p* < 0.05 versus Con. ^#^*p* < 0.05 versus CUP group. The western blot bands at the top show the target protein. The lower bands are internal controls (*β*-actin or *α*-tubulin).

To determine whether EA alters TRPV1 and associated molecules and regulate pain, we tested aforementioned pathways in SC tissue of the mice. We indicated that TRPV1 was expressed in the normal SC (Figure 3(a), 100.0%  ± 3.17%, *n* = 8), and that the expression level increased in chronic inflammatory pain in mice SC (Figure 3(a), 137.55%  ± 15.46%, *n* = 8). In particular, EA significantly reduced TRPV1 increase in SC (Figure 3(a), 104.23%  ± 3.84%, *n* = 8), and a similar result was observed in TRPV1^−/−^ mice (Figure 3(a), *n* = 8) but not in sham-operated group (Figure 3(a), 141.46%  ± 15.92%, *n* = 8). Then, we tested the expression levels of pPKA, pPI3K, and pPKC. We showed that pPKA, pPI3K, and pPKC were all increased in chronic inflammatory pain in mice SC (Figure 3(a), 153.78 ± 21.35%, 136.56 ± 11.51%, and 174.29 ± 17.34%, *n* = 8). This phenomenon is reversed by EA (Figure 3(a), 95.35 ± 5.83%, 102.73 ± 6.16%, and 104.31 ± 14.35%, *n* = 8) and in TRPV1 knockout mice (Figure 3(a), 94.34 ± 10.82%, 103.24 ± 5.68%, and 108.44 ± 10.59%, *n* = 8). A similar pattern was not found in the sham EA group (Figure 3(a), 135.24 ± 11.27%, 131.68 ± 10.98%, and 166.03 ± 23.24%, *n* = 8). We further showed that pERK, pp38, pJNK, and pAkt expression levels increased in SC of CFA-induced chronic inflammatory pain in mice (Figure 3(b), 132.4 ± 9.81, 123.85 ± 6.57%, 148.3 ± 10.76%, and 151.04 ± 16.12%, *n* = 8), which were further attenuated by EA (Figure 3(b), %, 104.3 ± 5.12, 103.42 ± 3.52%, 105.32 ± 5.82%, and 96.52 ± 13.83%, *n* = 8) and TRPV1 gene deletion treatment (Figure 3(b), 99.17 ± 7.18%, 103.65 ± 4.09%, 108.66 ± 5.16%, and 93.11 ± 10.49%, *n* = 8) but not in the sham group (Figure 3(b), 130.01 ± 9.84%, 128.64 ± 9.73%, 147.09 ± 9.02%, and 143.51 ± 12.81%, *n* = 8). Similar results were also observed in pmTOR expression (Figure 2(c), *n* = 8).

The expression levels of pCREB and pNK*κ*B increased in CFA-induced chronic inflammatory pain in mice (Figure 3(c), 165.26%  ± 21.54% and 153.85%  ± 12.51%, *n* = 8), which was reduced by EA (Figure 3(c), 102.2%  ± 17.53% and 94.58%  ± 12.46%, *n* = 8) and TRPV1 null mice (Figure 3(c), 103.86%  ± 20.41% and 96.53%  ± 5.44%, *n* = 8) but not in the sham-operated group (Figure 3(c), 159.02%  ± 13.4% and 142.18%  ± 20.9%, *n* = 8). The expression levels of Nav1.7 and Nav1.8 showed similar results (Figure 3(c), *n* = 8). Similar results were also observed for inflammatory factors such as GFAP, S100B, and RAGE (Figure 3(d), *n* = 8). The aforementioned results provide evidence that the TRPV1 signaling pathway is important in the central nerve system of mice with chronic inflammatory pain.

## 4. Discussion

TRPV1 is known to be involved in the perception of inflammatory and thermal pain, especially pain from heat, which exceeds 43°C [10]. The activation of TRPV1 can further initiate Ca^2+^ influx for neuronal depolarization [24, 25]. The downregulation of TRPV1 function results in insensitivity to high temperature stimulation, radial heat, and hot-plate tests [26]. Inflammatory mediators can induce hyperalgesia to activate TRPV1, suggesting their important roles in inflammatory pain [27]. Injection of TRPV1 antagonist capsazepine reduced thermal hyperalgesia in the inflammatory pain model [28, 29]. In inflammatory pain, proinflammatory mediators induce neuronal hyperactivity, initiating TRPV1 activation [27]. In the present study, we suggested that EA can reduce the overexpression of GFAP, S100B, and RAGE to reduce chronic inflammatory pain. The release of NGF can further bind the TrkA receptor and activate the PI3 kinase and Src kinase to phosphorylate TRPV1 [30]. TRPV1 is also regulated by phosphatidylinositol 4,5-bisphosphate, increasing the phosphorylation of TRPV1 by protein kinases [31–34]. Protease-activated receptor 2 is suggested to sensitize the TRPV1 receptors for thermal hyperalgesia by activating protein kinase A or protein kinase C*ε* signals [35]. Recent study, using a rat model of monoarthritis, showed that IL-1, IL-6, and TNF-*α* were activated after CFA injection [36]. Another study also indicated the relationship of inflammatory pain with TRPV1 and associated molecules in mice DRG and SC [17]. Lu et al. reported that the expression of TRPV1 and associated signaling pathways was increased after the CFA injection; the overexpression can be further reduced by EA treatment and TRPV1 gene deletion, suggesting that TRPV1 knockout mice are resistant to inflammatory pain [19]. Our data showed that these protein kinases increased in chronic pain model and can be further reduced by EA and TRPV1 gene deletions. The aforementioned mechanisms indicated that TRPV1 was involved in chronic inflammatory pain models and served as key receptors to sense mechanical and thermal pain.

Wu et al. indicated an abundance of TRPV1 in anatomical layers of ST36, especially in muscle layer. Immunofluorescence data also showed that TRPV1 is expressed in both neural and nonneural cells at ST36 acupoint. Injection of capsaicin, a TRPV1 agonist, into ST36 acupoints can relieve inflammatory pain as effectively as manual acupuncture treatment, replicating the analgesic effect of acupuncture [37]. Zhang et al. indicated that EA at ST36 acupoints could attenuate cancer-induced pain through attenuating TRPV1 expression in both mRNA and protein levels in DRG from tumor-bearing rats. They found that the injection of cancer cells could alleviate the paw withdrawal threshold and increase thermal hyperalgesia. The phenomenon could be further reversed by EA at the ST36 acupoints at 2 Hz low frequency [38]. Chen et al. documented that EA at bilateral ST36 acupoints can reduce mechanical and thermal hyperalgesia induced by injection of CFA or carrageenan into the plantar. They indicated that TRPV1 and TRPV4 increased in inflammatory pain and can be further attenuated by EA stimulation at the peripheral DRG neurons. These results not only indicated pain relief by EA but also suggested that EA could reduce the overexpression of TRPV1 and TRPV4 in small-to-large diameter neurons [39]. Paradoxically, patients receiving chronic opiates reportedly experience hyperalgesia. The hyperalgesia was accompanied with increased TTX-R sodium and TRPV1 channels in sensory neurons [40]. In the present study, we suggested that EA is beneficial for chronic inflammatory pain management by controlling TRPV1 overexpression.

**Figure 4:**
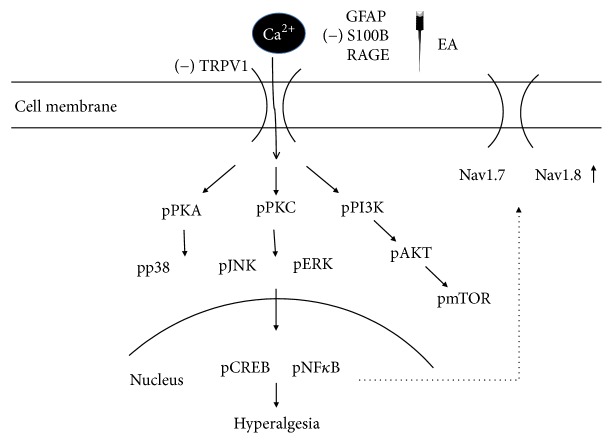
Schematic illustration of possible mechanisms of TRPV1 in CFA-induced chronic inflammatory pain in mice model. We showed that TRPV1 is an important receptor in mice chronic inflammatory pain model. The activation of TRPV1 increases the expression of pPKA, pPI3K, pPKC, pAkt, and pmTOR. Furthermore, pERK, pp38, pJNK, pNF*κ*B, and pCREB are also increased. Moreover, nociceptive Nav1.7 and Nav1.8 are increased for pain conduction in both peripheral DRG and central SC. Inflammatory factors such as GFAP, S100B, and RAGE are also involved in this process.

In the present study, our data suggested that EA can significantly reduce chronic inflammatory pain by downregulating the increased signaling of TRPV1 pathway from peripheral DRG to central SC. First, 2 Hz EA reduced CFA-induced chronic mechanical and thermal hyperalgesia. Second, the TRPV1 signaling pathway was increased in chronic inflammatory pain in mice DRG and further reversed by EA but not in the sham-operated group. Furthermore, similar results were also obtained by deleting the TRPV1 gene. Moreover, we showed that TRPV1 and downstream molecules were also altered in SC supporting the role for central sensitization. Taken together, these mechanisms provide a clear signal of TRPV1 and relevant molecules (Figure 4). These findings provide significant evidence to practice the clinical application of EA to treat chronic inflammatory pain.
